# The Role of Chemokines during Viral Infection of the CNS

**DOI:** 10.1371/journal.ppat.1000937

**Published:** 2010-07-29

**Authors:** Martin P. Hosking, Thomas E. Lane

**Affiliations:** 1 Department of Molecular Biology and Biochemistry, University of California, Irvine, California, United States of America; 2 Sue and Bill Gross Stem Cell Center, University of California, Irvine, California, United States of America; 3 Institute for Immunology, Infectious Diseases, and Vaccines, University of California, Irvine, California, United States of America; University of California San Francisco, United States of America

Viral infection of the central nervous system (CNS) poses unique challenges to the immune system with regards to controlling and eliminating the invading pathogen. These obstacles include the presence of a blood–brain barrier (BBB) that provides a physical and physiological barrier that is difficult for cells and molecules to cross, the absence of classic lymphatic drainage that may impair the generation of an adaptive immune response, and limited MHC class I or II expression on resident cells of the CNS, even during periods of neuroinflammation. In addition, the CNS is composed of a variety of highly specialized cells, many of which have limited renewal capacity, that represent potential targets of infection by numerous different viruses. Nonetheless, antigen-specific lymphocytes are ultimately able to accumulate within the CNS and contribute to defense by reducing or eliminating the invading viral pathogen. Alternatively, infiltration of activated cells of the immune system may be detrimental, as these cells can contribute to neuropathology that may result in long-term cellular damage or death. Understanding the mechanisms that govern leukocyte trafficking from the microvasculature into the CNS parenchyma is therefore critical for comprehending the molecular and cellular events that control neuroinflammation following infection by neurotropic viruses. Chemokines, small (8–10 kDa) proteins expressed by almost all nucleated cell types, are divided into four subfamilies based upon the number and spacing of conserved cysteine residues present within the amino terminus of the protein. Chemokine function is controlled through often promiscuous signaling via seven transmembrane G-protein-coupled receptors. While initially characterized as important in inflammation by targeting distinct leukocyte populations, chemokines are now considered critical mediators of a variety of biological processes, including development, tissue homeostasis, and coordinated immune responses during viral infection.

## Resident Cells of the CNS Secrete Chemokines in Response to Viral Infection

Chemokines are now recognized as critical regulators of leukocyte trafficking into the CNS. This leads to the inevitable questions, which cells are producing chemokines and how is this controlled? Numerous studies have revealed that resident cell populations of the CNS are able to synthesize and secrete a variety of chemokines. Astrocytes and microglia are the primary source of chemokines following infection with a wide range of neurotropic viruses, including the JHM strain of mouse hepatitis virus (JHMV), lymphocytic choriomeningitis virus (LCMV), Theiler's murine encephalitis virus (TMEV), herpes simplex virus 1 (HSV1), and human immunodeficiency virus (HIV) [1]–[4]. Neurons are also capable of secreting chemokines during HIV and West Nile virus (WNV) infection [5], [6], while endothelial cells express chemokines during simian immunodeficiency virus-induced encephalitis [7]. Both in vitro and in vivo studies have highlighted that CNS viral infection often results in distinct chemokine signature patterns. For example, Prehaud and colleagues have demonstrated that in vitro infection of neurons with rabies virus (RABV) results in robust production of chemokines, whereas HSV-1-infected neurons do not [8]. However, specific chemokines, e.g., CXCL10 and CCL5, are often expressed independently of either cellular tropism or viral genetics, suggesting that factor(s) either secreted in response to infection (such as type I interferon [IFN]) or utilized for viral recognition are shared between many neurotropic viruses. Toll-like receptors (TLRs) recognize both DNA and RNA and they are able to rapidly respond to viral infection, in part, by promoting chemokine gene expression. During TMEV infection, TLR2 and TLR3 cooperation leads to the expression of the macrophage chemoattractants CCL2 and CCL5 [3], while TLR2 and TLR9 mediate chemokine expression during HSV-1 infection [4], [9]. Type I IFNs regulate glial-derived chemokine expression in response to CNS infection with LCMV (*Traub* strain) and HSV-1 [2], [9]; however, this pathway is dispensable for expression of other chemokines, e.g., CCL2 following infection with JMHV [10]. Rather, JHMV viral proteins influence chemokine secretion through as yet undefined mechanisms [11], while the HIV-1 protein Nef influences neuronal chemokine secretion [5]. Moreover, WNV-infected cerebellar granule cell neurons readily secrete CXCL10 in vitro, while CXCL10 expression by WNV-infected cortical neurons is muted [12]. The consequence of this differential expression of CXCL10 is reflected in altered migration of defined inflammatory cells into the cerebellum at the expense of other WNV-infected CNS regions [12]. Collectively, these data illustrate that viral infection of the CNS by a wide variety of neurotropic viruses induces highly orchestrated and individual patterns of chemokine secretion by resident cells of the CNS, evoked by disparate pathways that converge into often overlapping profiles of inflammatory cell infiltration.

## Chemokines Regulate Immune Cell Access into the CNS

**Figure 1:**
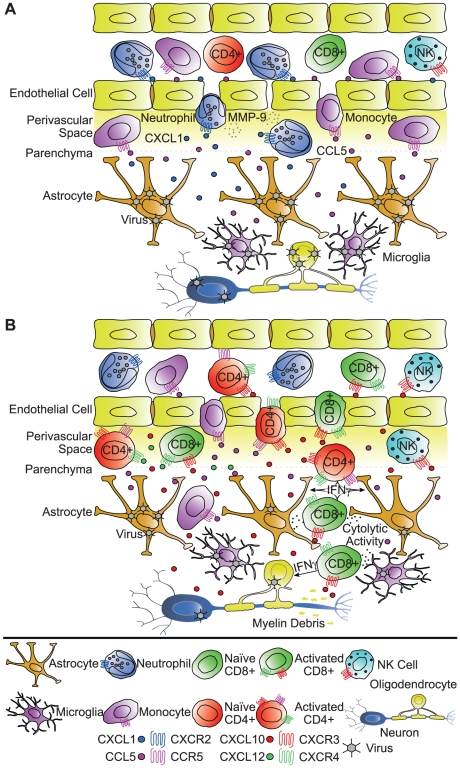
Functional roles of chemokines in response to viral infection of the CNS. Potential roles of chemokines in attracting innate immune cells (**A**) and lymphocytes (**B**) into the CNS during acute viral infection. The cartoons emphasize several key points derived from recent studies focusing on experimental infection with neurotropic viruses. (**A**) Early (days 1–3) after viral infection, activated and/or virally infected astrocytes, microglia, and endothelial cells secrete chemokines that serve to attract myeloid cells to the CNS. Among the earliest cells to respond to viral infection, neutrophils are recruited into the CNS by virtue of CXCR2 responding to ligands expressed within the CNS (e.g., CXCL1). Monocytes are also attracted into the CNS via the chemokine CCL5 and its receptor CCR5. Neutrophils and monocytes participate in the degradation of the blood–brain barrier (BBB), in part through the release of the matrix metalloproteinase MMP-9, and therefore ensure successive infiltration of virus-specific lymphocytes into the CNS. (**B**) During the acute stage of disease, astrocytes, microglia, neurons, and endothelial cells continue to secrete chemokines, serving to attract activated T lymphocytes, NK cells, and monocytes into the CNS. CD8+ and CD4+ T lymphocytes bearing the receptor CXCR3 and/or CCR5 are attracted by the chemokines CXCL10 and CCL5, respectively, and mediate viral control through direct cytolytic activity and/or cytokine secretion. CXCL12, which signals through CXCR4, may, however, sequester T lymphocytes within the perivascular space and regulate penetration of the parenchyma, thus inhibiting efficient viral clearance.

Signaling events that occur early following viral infection are often critical in dictating outcome. Recent studies have highlighted the importance of innate immune cells in contributing to a protective response, and we are just now learning how chemokines are involved in attracting these cells to the CNS. Infection of mice with neurotropic virus such as HSV-1 and JHMV results in the rapid accumulation of neutrophils to the CNS [13], [14]. Studies using the JHMV model system have provided insight into the functional relevance of neutrophil migration to the CNS, as these cells are required to contribute to the permeabilization of the BBB [14]. During JHMV infection, astrocyte- and endothelial-derived expression of ELR+ (glutamic acid-leucine-arginine) CXC chemokines, including CXCL1, attracts CXCR2-reactive neutrophils to the CNS [14]. Neutralization of this signaling axis specifically abrogates neutrophil infiltration, thereby preventing BBB degradation and the ensuing entry of protective JHMV-specific T lymphocytes [14]. In the absence of CXCR2 signaling, JHMV-infected mice experience higher viral loads and quickly succumb to infection, indicating that neutrophil targeting of the CNS is critical in host defense [14]. Conversely, McGavern and colleagues have suggested that during acute LCMV infection (*Armstrong* strain), cytotoxic T lymphocyte (CTL)-mediated chemokine gene expression contributes to fatal meningoencephalitis, in part, by attracting neutrophils and monocytes into the CNS, and this is associated with fatal vascular permeability and seizures, thus highlighting a detrimental role for neutrophils in response to viral infection [15]. In addition to enhancing the permeabilization of the BBB by recruiting neutrophils and monocytes, chemokines can also function as gatekeepers regulating leukocyte penetration into the parenchyma. Following WNV infection of the CNS, CXCL12 retains antigen-sensitized lymphocytes within the perivascular space. Antagonism of CXCR4, the receptor for CXCL12, enhances T lymphocyte entry into the CNS parenchyma, and this correlates with reduced WNV burden, enhanced survival, and limited neuropathology [16]. Thus, expression of chemokines early in response to infection with neurotropic viruses aids in effective host defense by promoting vascular permeability and regulating parenchymal lymphocyte infiltration (Figure 1A). However, it should be emphasized that the consequences of BBB degradation can vary from efficient viral clearance to fatal encephalitis and seizures, depending upon the virus and the route of infection.

## Chemokines and Neuroprotection during Viral Infection

Although early signaling events are clearly important for host defense during viral infection, the infiltration and anti-viral activity of T lymphocytes are requisite for viral clearance and survival. CXCL10, which is prominently expressed within the CNS during many viral infections [1], [17], functions to attract activated T lymphocytes bearing the receptor CXCR3. Neutralization or genetic silencing of CXCL10 following infection with HSV, JHMV, and WNV dramatically reduces T cell trafficking into the CNS, thus preventing efficient viral control and often resulting in poor resolution [6], [18], [19]. In addition to attracting T lymphocytes, the CXCR3 ligands CXCL10 and CXCL9 also attract natural killer (NK) cells during JHMV infection [20], [21]; however, their role in viral clearance remains unclear. The macrophage and T lymphocyte chemokine CCL5, or one of its receptors, CCR5, also promotes leukocyte trafficking into the CNS and subsequent viral control during JHMV infection and WNV-induced encephalitis [22], [23]. The clinical relevance of this observation was revealed when homozygosity for the defective human CCR5 allele (CCR5Δ32) was associated with an increased risk for symptomatic WNV infection [24]. Collectively, these data demonstrated that chemokine expression during viral infection promotes the generation and infiltration of immune effector cells necessary for quelling viral replication (Figure 1B).

## Chemokines and Neuropathology following Viral Infection

A potential consequence of chemokine secretion and the subsequent accumulation of leukocytes within the CNS, while important for viral control in many instances, is the development of neuropathology. For example, the fatal meningoencephalitis induced by LCMV infection is mediated by infiltration of virus-specific CTLs that promote subsequent myeloid cell and leukocyte entry [15], [25]. During infection with LCMV (*Traub*), genetic silencing of CXCL10 or its receptor CXCR3 reduces the infiltration of CD8+ T cells, conferring either partial or near complete protection from immunopathology and death [26], [27]. However, CXCL10 remains dispensable for T cell infiltration or the development of fatal inflammation during infection with LCMV (*Armstrong*) [28], further highlighting underlying differences in viral strains and chemokine utilization with regards to disease outcome. During JHMV infection, sustained CXCL10 and CCL5 expression leads to continuing immune cell infiltration that manifests an immune-mediated demyelinating disease. Neutralization of either chemokine during persistent JHMV infection abrogates the immune infiltration and greatly reduces both disease severity and demyelination [29], [30]. In addition to attracting inflammatory cells that contribute to neuropathology, CXCL10, which is chronically expressed within the brains of patients suffering from HIV-associated neurological disorders, can directly induce neuronal cell death [5]. In addition, proteolytically cleaved CXCL12, which is also detectable within the brains of HIV-1-infected patients, is capable of inducing neurotoxicity and apoptosis [31]. Although beyond the scope of this review, extensive work has focused upon the direct and indirect roles of the chemokine receptors CXCR4 and CCR5 (and their associated ligands) in contributing to HIV-associated dementia (reviewed in [32], [33]). Therefore, chemokines are critical mediators of neuropathology during viral infections of the CNS, either by attracting pathogenic inflammatory cells or directly mediating neurotoxicity and cell death.

## Conclusions

From this brief review, it is evident that the biological roles of chemokines in host defense and/or disease in response to viral infection of the CNS are constantly evolving. An emerging picture has developed that indicates that chemokines and their receptors are intimately involved in generation of effective host responses to viral infections within the CNS. Paradoxically, chemokine expression has also been associated with neuropathology. Thus, chemokines and/or chemokine receptors are potentially relevant targets for treating various viral-induced neuropathies by dampening specific biological functions associated with disease. Recent evidence has emerged implicating chemokines, specifically CXCR4 and CXCL12, as important mediators of neurogenesis [34]; thus, chemokines produced during viral infections may influence neural precursor cell function and therefore influence recovery and repair. We can only look forward to future research that will undoubtedly uncover new and exciting roles for the chemokines in host defense, disease, and recovery within the context of the virally infected CNS.
